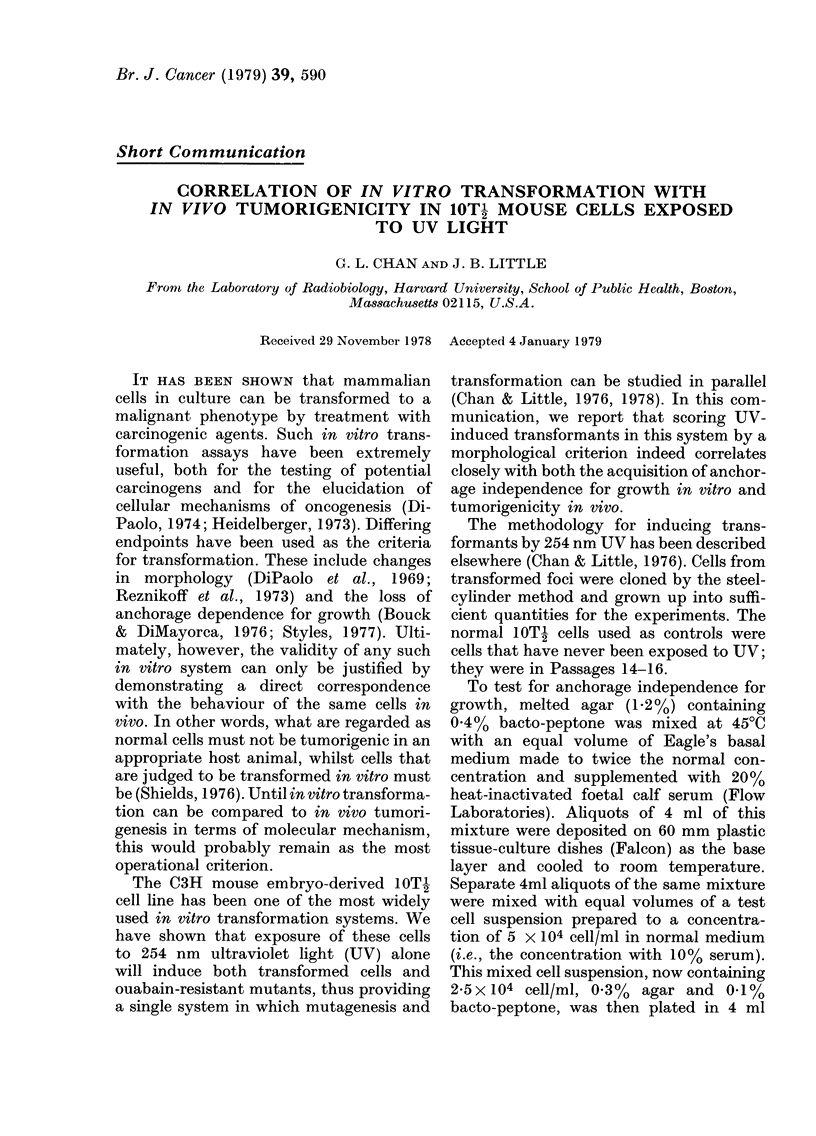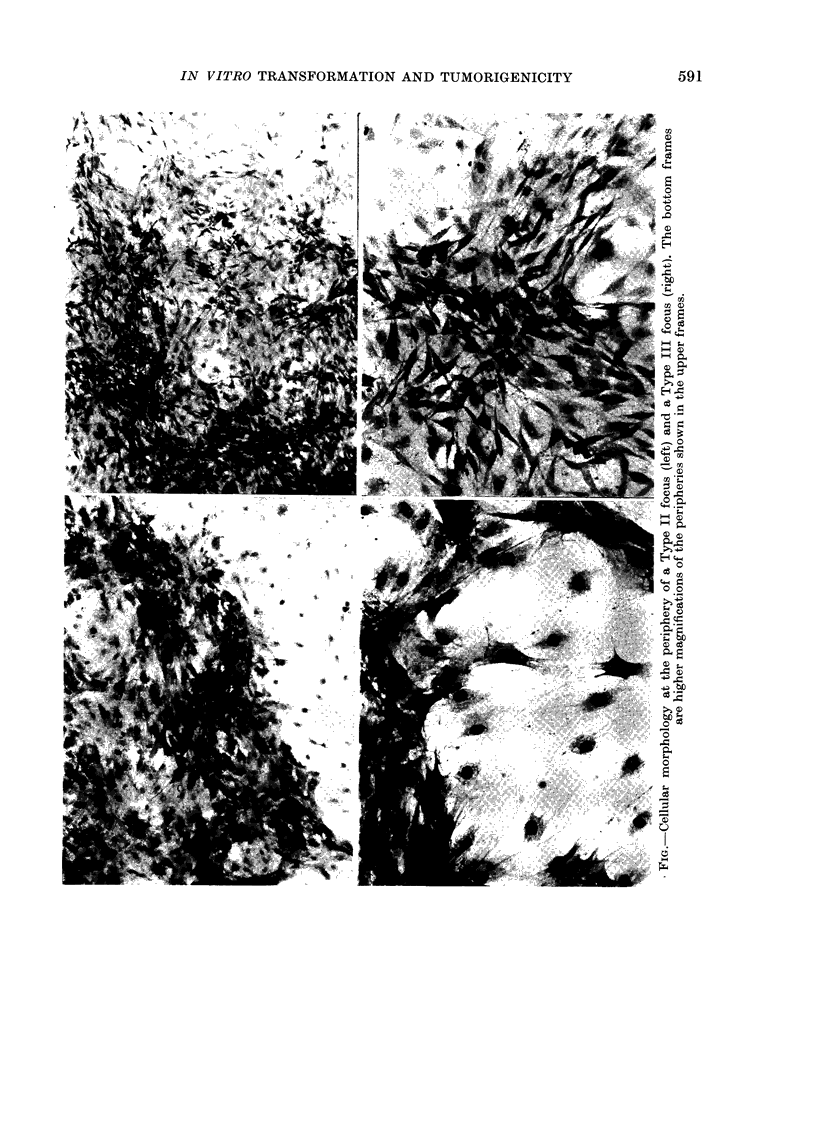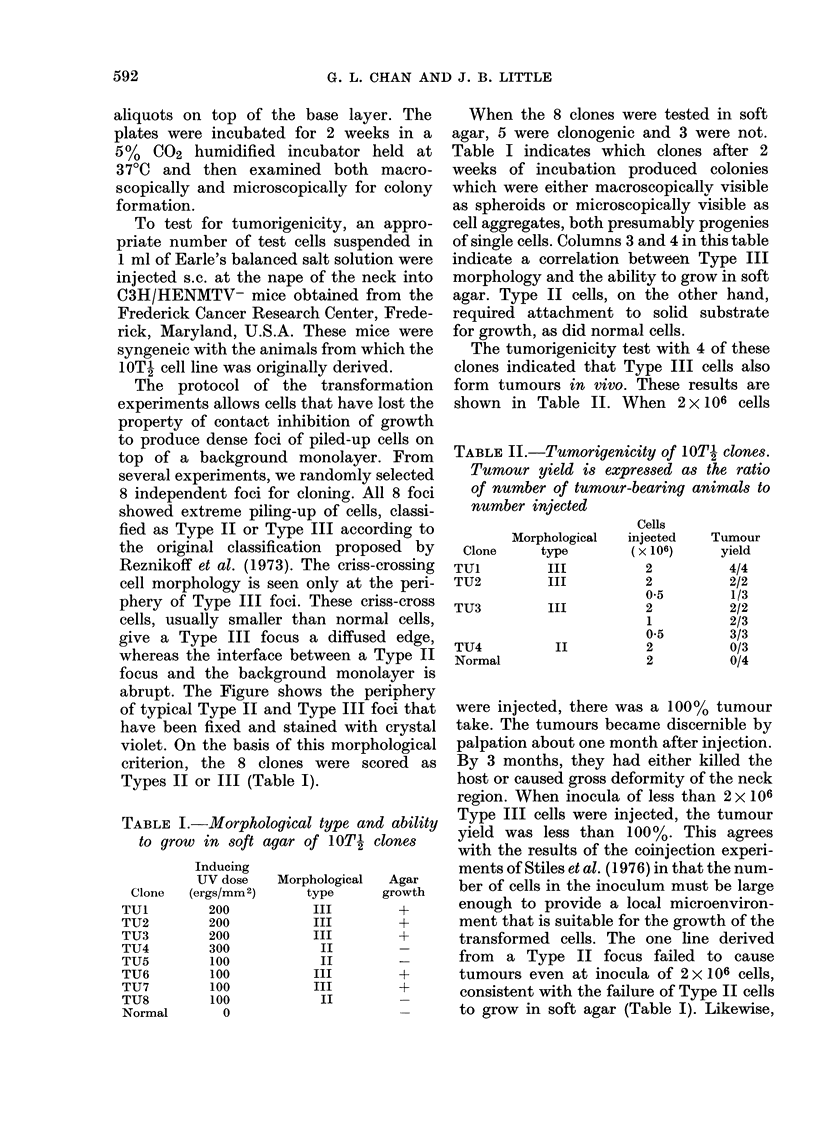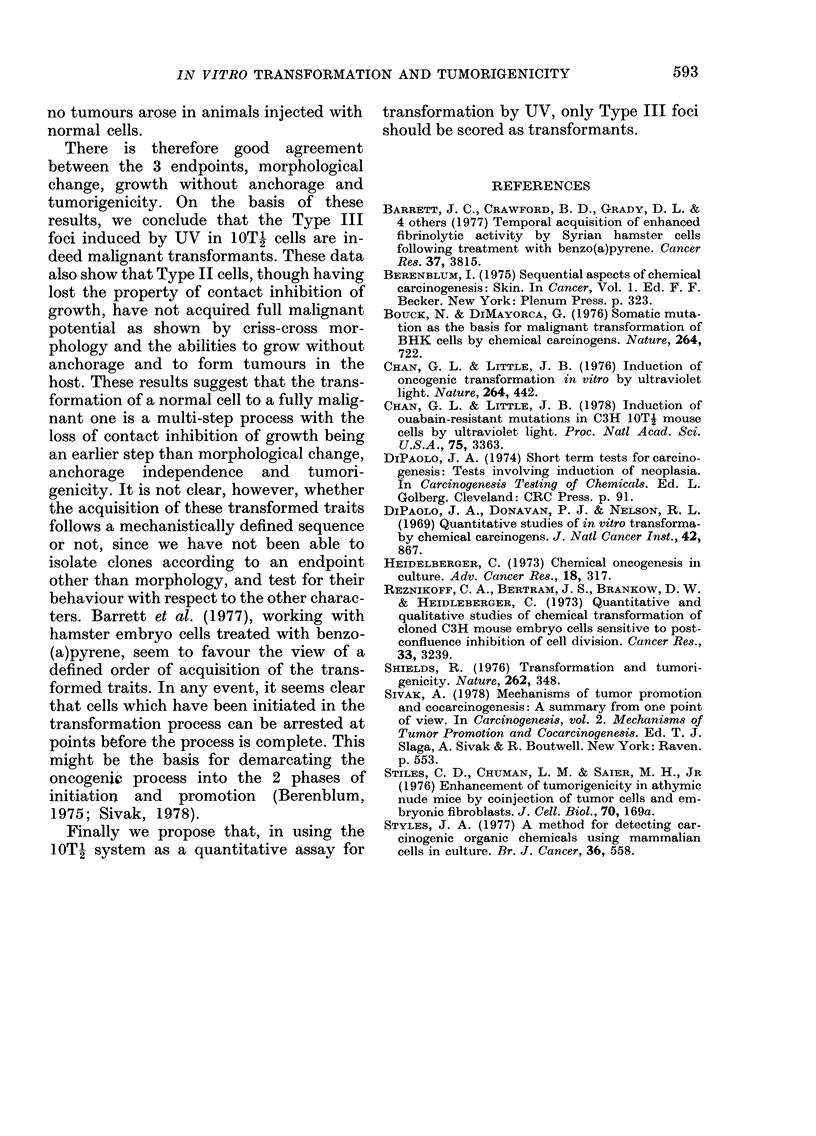# Correlation of in vitro transformation with in vivo tumorigenicity in 10T1/2 mouse cells exposed to UV light.

**DOI:** 10.1038/bjc.1979.107

**Published:** 1979-05

**Authors:** G. L. Chan, J. B. Little

## Abstract

**Images:**


*Br. J. Cancer* (1979) **39**, 590

***Short Communication***

### CORRELATION OF *IN VITRO* TRANSFORMATION WITH *IN VIVO* TUMORIGENICITY IN 10T½ MOUSE CELLS EXPOSED TO UV LIGHT

G. L. CHAN AND J. B. LITTLE

*From the Laboratory of Radiobiology, Harvard University, School of Public Health, Boston, Massachusetts* 02115, *U.S.A.*

Received 29 November 1978 Accepted 4 January 1979

IT HAS BEEN SHOWN that mammalian cells in culture can be transformed to a malignant phenotype by treatment with carcinogenic agents. Such *in vitro* transformation assays have been extremely useful, both for the testing of potential carcinogens and for the elucidation of cellular mechanisms of oncogenesis (DiPaolo, 1974; Heidelberger, 1973). Differing endpoints have been used as the criteria for transformation. These include changes in morphology (DiPaolo *et al.*, 1969; Reznikoff *et al.*, 1973) and the loss of anchorage dependence for growth (Bouck & DiMayorca, 1976; Styles, 1977). Ultimately, however, the validity of any such *in vitro* system can only be justified by demonstrating a direct correspondence with the behaviour of the same cells *in vivo*. In other words, what are regarded as normal cells must not be tumorigenic in an appropriate host animal, whilst cells that are judged to be transformed *in vitro* must be (Shields, 1976). Until *in vitro* transformation can be compared to *in vivo* tumorigenesis in terms of molecular mechanism, this would probably remain as the most operational criterion.

The C3H mouse embryo-derived 10T½ cell line has been one of the most widely used *in vitro* transformation systems. We have shown that exposure of these cells to 254 nm ultraviolet light (UV) alone will induce both transformed cells and ouabain-resistant mutants, thus providing a single system in which mutagenesis and transformation can be studied in parallel (Chan & Little, 1976, 1978). In this communication, we report that scoring UV-induced transformants in this system by a morphological criterion indeed correlates closely with both the acquisition of anchorage independence for growth *in vitro* and tumorigenicity *in vivo*.

The methodology for inducing transformants by 254 nm UV has been described elsewhere (Chan & Little, 1976). Cells from transformed foci were cloned by the steel-cylinder method and grown up into sufficient quantities for the experiments. The normal 10T½ cells used as controls were cells that have never been exposed to UV; they were in Passages 14–16.

To test for anchorage independence for growth, melted agar (1·2%) containing 0·4% bacto-peptone was mixed at 45°C with an equal volume of Eagle's basal medium made to twice the normal concentration and supplemented with 20% heat-inactivated foetal calf serum (Flow Laboratories). Aliquots of 4 ml of this mixture were deposited on 60 mm plastic tissue-culture dishes (Falcon) as the base layer and cooled to room temperature. Separate 4ml aliquots of the same mixture were mixed with equal volumes of a test cell suspension prepared to a concentration of $5 \times 10^4$ cell/ml in normal medium (*i.e.*, the concentration with 10% serum). This mixed cell suspension, now containing $2{\cdot}5 \times 10^4$ cell/ml, 0·3% agar and 0·1% bacto-peptone, was then plated in 4 ml

FIG.—Cellular morphology at the periphery of a Type II focus (left) and a Type III focus (right). The bottom frames are higher magnifications of the peripheries shown in the upper frames.

aliquots on top of the base layer. The plates were incubated for 2 weeks in a 5% $CO_2$ humidified incubator held at 37°C and then examined both macroscopically and microscopically for colony formation.

To test for tumorigenicity, an appropriate number of test cells suspended in 1 ml of Earle's balanced salt solution were injected s.c. at the nape of the neck into C3H/HENMTV$^-$ mice obtained from the Frederick Cancer Research Center, Frederick, Maryland, U.S.A. These mice were syngeneic with the animals from which the 10T½ cell line was originally derived.

The protocol of the transformation experiments allows cells that have lost the property of contact inhibition of growth to produce dense foci of piled-up cells on top of a background monolayer. From several experiments, we randomly selected 8 independent foci for cloning. All 8 foci showed extreme piling-up of cells, classified as Type II or Type III according to the original classification proposed by Reznikoff *et al.* (1973). The criss-crossing cell morphology is seen only at the periphery of Type III foci. These criss-cross cells, usually smaller than normal cells, give a Type III focus a diffused edge, whereas the interface between a Type II focus and the background monolayer is abrupt. The Figure shows the periphery of typical Type II and Type III foci that have been fixed and stained with crystal violet. On the basis of this morphological criterion, the 8 clones were scored as Types II or III (Table I).

TABLE I.—*Morphological type and ability to grow in soft agar of 10T½ clones*

| Clone | Inducing UV dose (ergs/mm$^2$) | Morphological type | Agar growth |
|---|---|---|---|
| TU1 | 200 | III | + |
| TU2 | 200 | III | + |
| TU3 | 200 | III | + |
| TU4 | 300 | II | − |
| TU5 | 100 | II | − |
| TU6 | 100 | III | + |
| TU7 | 100 | III | + |
| TU8 | 100 | II | − |
| Normal | 0 | | − |

When the 8 clones were tested in soft agar, 5 were clonogenic and 3 were not. Table I indicates which clones after 2 weeks of incubation produced colonies which were either macroscopically visible as spheroids or microscopically visible as cell aggregates, both presumably progenies of single cells. Columns 3 and 4 in this table indicate a correlation between Type III morphology and the ability to grow in soft agar. Type II cells, on the other hand, required attachment to solid substrate for growth, as did normal cells.

The tumorigenicity test with 4 of these clones indicated that Type III cells also form tumours *in vivo*. These results are shown in Table II. When $2\times10^6$ cells were injected, there was a 100% tumour take. The tumours became discernible by palpation about one month after injection. By 3 months, they had either killed the host or caused gross deformity of the neck region. When inocula of less than $2\times10^6$ Type III cells were injected, the tumour yield was less than 100%. This agrees with the results of the coinjection experiments of Stiles *et al.* (1976) in that the number of cells in the inoculum must be large enough to provide a local microenvironment that is suitable for the growth of the transformed cells. The one line derived from a Type II focus failed to cause tumours even at inocula of $2\times10^6$ cells, consistent with the failure of Type II cells to grow in soft agar (Table I). Likewise,

TABLE II.—*Tumorigenicity of 10T½ clones. Tumour yield is expressed as the ratio of number of tumour-bearing animals to number injected*

| Clone | Morphological type | Cells injected ($\times10^6$) | Tumour yield |
|---|---|---|---|
| TU1 | III | 2 | 4/4 |
| TU2 | III | 2 | 2/2 |
| | | 0·5 | 1/3 |
| TU3 | III | 2 | 2/2 |
| | | 1 | 2/3 |
| | | 0·5 | 3/3 |
| TU4 | II | 2 | 0/3 |
| Normal | | 2 | 0/4 |

no tumours arose in animals injected with normal cells.

There is therefore good agreement between the 3 endpoints, morphological change, growth without anchorage and tumorigenicity. On the basis of these results, we conclude that the Type III foci induced by UV in $10T\frac{1}{2}$ cells are indeed malignant transformants. These data also show that Type II cells, though having lost the property of contact inhibition of growth, have not acquired full malignant potential as shown by criss-cross morphology and the abilities to grow without anchorage and to form tumours in the host. These results suggest that the transformation of a normal cell to a fully malignant one is a multi-step process with the loss of contact inhibition of growth being an earlier step than morphological change, anchorage independence and tumorigenicity. It is not clear, however, whether the acquisition of these transformed traits follows a mechanistically defined sequence or not, since we have not been able to isolate clones according to an endpoint other than morphology, and test for their behaviour with respect to the other characters. Barrett *et al.* (1977), working with hamster embryo cells treated with benzo-(a)pyrene, seem to favour the view of a defined order of acquisition of the transformed traits. In any event, it seems clear that cells which have been initiated in the transformation process can be arrested at points before the process is complete. This might be the basis for demarcating the oncogenic process into the 2 phases of initiation and promotion (Berenblum, 1975; Sivak, 1978).

Finally we propose that, in using the $10T\frac{1}{2}$ system as a quantitative assay for transformation by UV, only Type III foci should be scored as transformants.

### REFERENCES

BARRETT, J. C., CRAWFORD, B. D., GRADY, D. L. & 4 others (1977) Temporal acquisition of enhanced fibrinolytic activity by Syrian hamster cells following treatment with benzo(a)pyrene. *Cancer Res.* **37**, 3815.

BERENBLUM, I. (1975) Sequential aspects of chemical carcinogenesis: Skin. In *Cancer*, Vol. 1. Ed. F. F. Becker. New York: Plenum Press. p. 323.

BOUCK, N. & DIMAYORCA, G. (1976) Somatic mutation as the basis for malignant transformation of BHK cells by chemical carcinogens. *Nature*, **264**, 722.

CHAN, G. L. & LITTLE, J. B. (1976) Induction of oncogenic transformation *in vitro* by ultraviolet light. *Nature*, **264**, 442.

CHAN, G. L. & LITTLE, J. B. (1978) Induction of ouabain-resistant mutations in C3H $10T\frac{1}{2}$ mouse cells by ultraviolet light. *Proc. Natl Acad. Sci. U.S.A.*, **75**, 3363.

DIPAOLO, J. A. (1974) Short term tests for carcinogenesis: Tests involving induction of neoplasia. In *Carcinogenesis Testing of Chemicals*. Ed. L. Golberg. Cleveland: CRC Press. p. 91.

DIPAOLO, J. A., DONAVAN, P. J. & NELSON, R. L. (1969) Quantitative studies of *in vitro* transforma-by chemical carcinogens. *J. Natl Cancer Inst.*, **42**, 867.

HEIDELBERGER, C. (1973) Chemical oncogenesis in culture. *Adv. Cancer Res.*, **18**, 317.

REZNIKOFF, C. A., BERTRAM, J. S., BRANKOW, D. W. & HEIDLEBERGER, C. (1973) Quantitative and qualitative studies of chemical transformation of cloned C3H mouse embryo cells sensitive to post-confluence inhibition of cell division. *Cancer Res.*, **33**, 3239.

SHIELDS, R. (1976) Transformation and tumorigenicity. *Nature*, **262**, 348.

SIVAK, A. (1978) Mechanisms of tumor promotion and cocarcinogenesis: A summary from one point of view. In *Carcinogenesis, vol. 2. Mechanisms of Tumor Promotion and Cocarcinogenesis.* Ed. T. J. Slaga, A. Sivak & R. Boutwell. New York: Raven. p. 553.

STILES, C. D., CHUMAN, L. M. & SAIER, M. H., JR (1976) Enhancement of tumorigenicity in athymic nude mice by coinjection of tumor cells and embryonic fibroblasts. *J. Cell. Biol.*, **70**, 169*a*.

STYLES, J. A. (1977) A method for detecting carcinogenic organic chemicals using mammalian cells in culture. *Br. J. Cancer*, **36**, 558.